# Identification and Quantitation of Flavanols and Proanthocyanidins in Foods: How Good are the Datas?

**DOI:** 10.1080/10446670410001722177

**Published:** 2005-03

**Authors:** Mark A. Kelm, John F. Hammerstone, Harold H. Schmitz

**Affiliations:** 1 Analytical and Applied Sciences Group Masterfoods, Incorporated 800 High Street Hackettstown NJ 07840 USA; 2 Mars, Incorporated McLean VA 22101 USA

## Abstract

Evidence suggesting that dietary polyphenols, flavanols, and proanthocyanidins in particular offer significant cardiovascular health benefits is rapidly increasing. Accordingly, reliable and accurate methods are needed to provide qualitative and quantitative food composition data necessary for high quality epidemiological and clinical research. Measurements for flavonoids and proanthocyanidins have employed a range of analytical techniques, with various colorimetric assays still being popular for estimating total polyphenolic content in foods and other biological samples despite advances made with more sophisticated analyses. More crudely, estimations of polyphenol content as well as antioxidant activity are also reported with values relating to radical scavenging activity. High-performance liquid chromatography (HPLC) is the method of choice for quantitative analysis of individual polyphenols such as flavanols and proanthocyanidins. Qualitative information regarding proanthocyanidin structure has been determined by chemical methods such as thiolysis and by HPLC-mass spectrometry (MS) techniques at present. The lack of appropriate standards is the single most important factor that limits the aforementioned analyses. However, with ever expanding research in the arena of flavanols, proanthocyanidins, and health and the importance of their future inclusion in food composition databases, the need for standards becomes more critical. At present, sufficiently well-characterized standard material is available for selective flavanols and proanthocyanidins, and construction of at least a limited food composition database is feasible.


*Clinical & Developmental Immunology*, March 2005; 12(1): 35–41

Taylor & Francis

Taylor & Francis Group

### Identification and quantitation of flavanols and proanthocyanidins in foods: How good are the datas?

MARK A. KELM[1], JOHN F. HAMMERSTONE[1], & HAROLD H. SCHMITZ[2]

[1]*Analytical and Applied Sciences Group, Masterfoods, Incorporated, 800 High Street, Hackettstown, NJ 07840, USA, and* [2]*Mars, Incorporated, McLean, VA 22101, USA*

**Abstract**

Evidence suggesting that dietary polyphenols, flavanols, and proanthocyanidins in particular offer significant cardiovascular health benefits is rapidly increasing. Accordingly, reliable and accurate methods are needed to provide qualitative and quantitative food composition data necessary for high quality epidemiological and clinical research. Measurements for flavonoids and proanthocyanidins have employed a range of analytical techniques, with various colorimetric assays still being popular for estimating total polyphenolic content in foods and other biological samples despite advances made with more sophisticated analyses. More crudely, estimations of polyphenol content as well as antioxidant activity are also reported with values relating to radical scavenging activity. High-performance liquid chromatography (HPLC) is the method of choice for quantitative analysis of individual polyphenols such as flavanols and proanthocyanidins. Qualitative information regarding proanthocyanidin structure has been determined by chemical methods such as thiolysis and by HPLC–mass spectrometry (MS) techniques at present. The lack of appropriate standards is the single most important factor that limits the aforementioned analyses. However, with ever expanding research in the arena of flavanols, proanthocyanidins, and health and the importance of their future inclusion in food composition databases, the need for standards becomes more critical. At present, sufficiently well-characterized standard material is available for selective flavanols and proanthocyanidins, and construction of at least a limited food composition database is feasible.

**Keywords:** *Analytical standards, antioxidant, cardiovascular, database, flavanol, flavonoid*

### Introduction

Numerous studies have linked the consumption of plant foods with improved health status and reduced risk of chronic disease (Hertog et al. 1995, Santo-Buelga and Scalbert 2001, Rice-Evans et al. 2001, Knekt et al. 2002). Concurrently, advances in separation science, biology, and chemistry have catapulted the inter-disciplinary fields of pharmacognosy and natural products chemistry research to yield a wealth of information about many classes of naturally occurring dietary phytochemicals (Dawson 2000, Drewnowski and Gomez-Carneros 2000, Bramley, 2002). This improved understanding of food composition has provided the opportunity to better understand why some plant foods may offer important health benefits beyond those commonly associated with essential nutrients. Among the most commonly studied plant derived compounds have been the polyphenols, which in part, is due to their ubiquity throughout the plant kingdom. More recently, the potential to prevent the onset of disease (i.e. cancer, cardiovascular) by consuming foods rich in polyphenols has done much to encourage research endeavors in flavonoids and related compounds. A brief overview and critique of current methods of analysis for biologically relevant flavonoids; $C_6-C_3-C_6$ (**Figure** 1) and to a greater extent, proanthocyanidins; $(C_6-C_3-C_6)_n$ (**Figure** 2) will be the focus of this manuscript. Specifically, methods of quantification, structural characterization and antioxidant activity assessment will be addressed. In addition, the importance of analytical methodologies

Correspondence: M. A. Kelm, Analytical and Applied Sciences Group, Masterfoods, Incorporated, 800 High Street, Hackettstown, NJ 07840, USA. Tel: 1 908 850 2051. Fax: 1 908 850 2697. E-mail: mark.kelm@effem.com

ISSN 1740-2522 print/ISSN 1740-2530 online © 2005 Taylor & Francis Group Ltd.
DOI: 10.1080/10446670410001722177

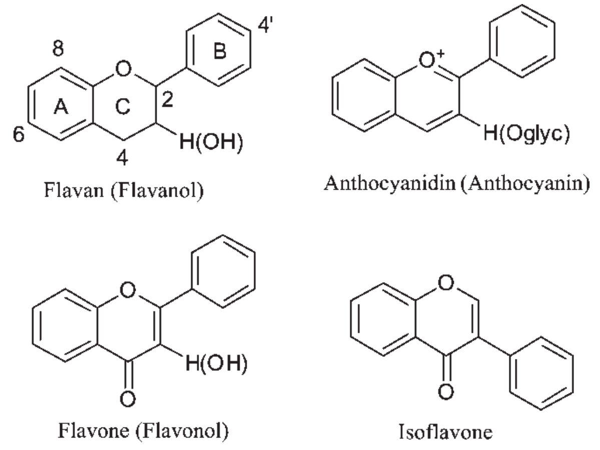

Figure 1. Major flavonoid subclasses found in foods.

for food and nutritional composition data, and epidemiological research will be discussed as well as improvement of future analytical data.

### Chemical methods of analysis

### *Colorimetric methods*

Colorimetric assays utilize reagents that react with phenolics to form colored products that are readily quantifiable by absorption measurements. These methods are of great utility for screening of plant materials for phenolics and as a way to measure gross phenolic content. Additionally, qualitative information regarding nature of phenolic structure is obtainable by methods described below. A very thorough description and application of the various colorimetric methods are available online (Hagerman 2002).

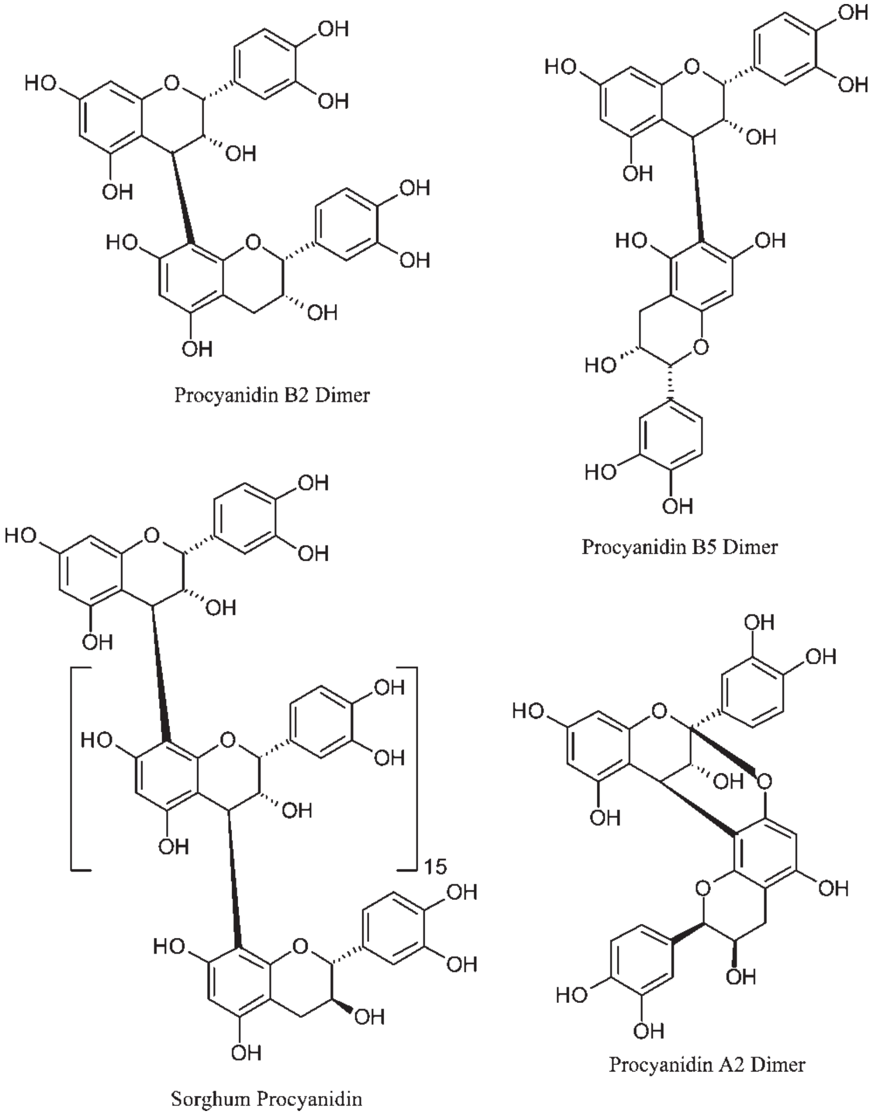

Figure 2. Examples of common proanthocyanidins found in foods. Note linkage differences of B- and A-type dimers.

Prussian-blue and Folin methods (Price and Butler 1977, Swain and Hillis 1959, Graham 1992) and variations thereof are widely used methods to screen plant material for phenolics. These methods are based on redox chemistry and in the case of Prussian-blue, involve treatment with ferric chloride ($FeCl_3$). Subsequent oxidation of phenolic compounds, formation of Prussian-blue, and absorbance measurements (700 nm) is used for quantification. These assays are standardized typically with simple phenolics such as gallic acid. However, in the case of oligomeric proanthocyanidins, a different response typically occurs. Therefore, data obtained cannot be expressed on a mass basis rather in terms of "gallic acid equivalents". Furthermore, it should be emphasized that these methods are non-specific in that they measure total phenolic content (i.e. phenolic acids, flavonoids, proanthocyanidins, etc.).

Other methods for detection of proanthocyanidins (condensed tannins) include; vanillin, *p*-dimethylaminocinnamaldehyde (DMACA), and acid–butanol. The variations of these methods have been used to provide qualitative (i.e. estimation of molecular weights) and quantitative data (Hagerman 2002).

The vanillin assay or reaction has been used extensively to quantify proanthocyanidin content of various plant materials, whereby vanillin adducts is measured at 500 nm. Chemically similar to the vanillin assay, a high-performance liquid chromatography (HPLC) colorimetric post-column derivatization by DMACA procedure has been applied to catechin (Treutter 1990). DMACA derivatization has also been applied to proanthocyanidins (de Pascual-Teresa et al. 1998). DMACA condensates are detected at 640 nm thus offering greater selectivity over conventional UV detector settings. Problems associated with vanillin and DMACA assays are due to the fact that both measure flavonols, dihydrochalcones and proanthocyanidins. Other problems with the assay lie with the use of catechin as a standard. Differences in color formation between catechin standards and polymers are due to differences in reaction rates. 5-deoxy proanthocyanidins are under represented because vanillin reacts more so with meta-substituted flavonoids.

Proanthocyanidin estimates are determined by acid–butanol method (a.k.a. Bates-Smith and Porter methods) which oxidatively cleaves interflavanoid bonds to yield anthocyanidins, which are subsequently quantitated at 550 nm. Proanthocyanidin oligomeric degrees of polymerization are determined by addition of phloroglucinol following cleavage by acid–butanol, thereby yielding adducts and terminal units which are then analyzed by HPLC. Removal of chlorophyll or other pigments are often necessary to minimize interference. Reaction of acid–butanol with 5-deoxy proanthocyanidins is similar to vanillin assay. However, the acid–butanol method tends to be quite reliable and less problematic than vanillin based methods.

### *Thiolysis*

Degrees of polymerization and nature of flavan-3-ol units of proanthocyanidins is achievable by thiolysis. Thiolysis involves treatment of the proanthocyanidin with benzyl mercaptan or toluene α-thiol in acidic medium to yield corresponding benzyl-or tolyl thioethers, respectively, whereas terminal units are released as free flavon-3-ols. Thiolysis products are distinguished by reverse phase (RP) chromatography by UV detection at 280 nm. The average degrees of polymerization are determined by calculating the ratio between total units (terminal units plus thioethers) and terminal units. Sodium cyanoborohydride has been used to reductively cleave a type of proanthocyanidins (Lou et al. 1999). Thiolysis of polymeric procyanidins (DP $> 10$) by Gu et al. (2002) indicated presence of both epicatechin and catechin as terminal units and epicatechin extension units in cocoa, sorghum bran, blueberry, and cranberry. Cranberry also contained A-types in roughly half of its terminal units. A-type proanthocyanidins (doubly linked, see **Figure** 2) are resistant to degradation by thiolysis (Kerchesy and Hemingway 1986, Hagerman 2002).

Usefulness of thiolytic methods are limited by epimerization of flavan-3-ol units (Porter 1994, Matthews et al. 1997) thus making it difficult to estimate the composition of terminal units. Poor yields due to reaction product instability, reactions with non-proanthocyanidin compounds, and side reactions also contribute negatively to the utility of thiolytic methods (Matthews et al. 1997).

### Instrumental methods of analysis

### *HPLC and HPLC–MS—flavonoids*

Merken and Beecher (2000) have produced a comprehensive review on the HPLC of food flavonoids. HPLC methods for anthocyanidins, flavones, flavanones, flavonols, and flavonol glycosides are tabulated. Typically, flavonoids are subjected to RP chromatography and are detected between 245 and 350 nm. Generally the HPLC analysis of glycosylated flavonoids is quite difficult due to co-elution and poor resolution; however, Merken et al. (2001) reports a simplified HPLC analysis of flavonoids by subjecting food samples to acid hydrolysis. Acidic hydrolysis converts flavonoid glycosides into their corresponding aglycones, which were then subjected to HPLC analysis and quantification. However, Merken observed destruction of flavanols and flavanones during acid hydrolysis. A better non-destructive approach to the analysis of glycosylated flavonoids would be to use HPLC/MS. Co-elution is less of an issue when one can differentiate between masses. Grayer et al. (2000) demonstrated the utility of APCI LC–MS in the characterization of flavonoid glycosides from *Ocimum gratissium*. Tea catechins were analyzed using the same technique (Zeeb et al. 2000). In both papers, fingerprints composed of molecular ions and diagnostic fragments (i.e. retro Diels-Alder) for individual compounds aided in their unambiguous identification. Anthocyanins (anthocyanidin glycosides) were identified and quantified by LC/ES–MS and HPLC, respectively, in a variety dried berry powders (Chandra et al. 2001). Quantification of individual anthocyanins was determined from external standard (cyanidin-3-glycoside chloride) curves and molecular weight correction factors. Estimated measurements for total anthocyanidins are measured at 520 nm using average extinction coefficients (Shahidi and Naczk 1995). The analysis of intact molecules would be more biologically relevant especially when tabulating flavonoid values for foods in nutrient databases.

### *HPLC and HPLC–MS—proanthocyanidins*

For proanthocyanidins in foods, analysis by RP chromatography often has been the primary method of choice. HPLC–UV quantitative analysis of proanthocyanidins in foods is typically carried out at 280 nm. However, UV detection is not specific for proanthocyanidins relative to other polyphenolic compounds. Fluorescence detection (excitation 276 and emission 316 nm) offers increased sensitivity and selectivity to procyanidins (Lazarus et al. 1999) but not for prodelphinidins and gallic acid esters. RP C18 columns have been used to fractionate monomers to trimers in the Spanish diet (de Pascual-Teresa et al. 2000) to trimers in apple (Yanagida et al. 2000), to tetramers in wine (Carando et al. 1999) and grape seed (Fuleki 1997, Peng et al. 2001). Proanthocyanidin oligomers do separate based on their degree of polymerization (monomers through tetramers) and as individual compounds however, order of elution is not in accordance with molecular size. Furthermore, analysis beyond tetramers has not been achievable with RP chromatography because of retention time overlap and co-elution of higher oligomeric isomers (Guyot et al. 1997). These problems are largely overcome by RP–HPLC–MS experiments where mass differentiation of cocoa procyanidin monomers through hexamers was demonstrated (Wollgast et al. 2001).

Early separations of proanthocyanidins by normal phase chromatography were met with limited success but over time more understanding came about for example, increases in retention times were found to correspond with increasing degrees of polymerization (Wilson 1979). In the case of procyanidins, normal phase HPLC separation is based on hydrogen bonding interactions between silica hydroxyls with the larger oligomers having more extensive interactions and thus longer retention times (Waterhouse et al. 2000). Rigaud et al. (1993) achieved the first successful separation of cocao bean procyanidins on a silica column by elution of dichloromethane–methanol–formic acid. Preparative and analytical separation of apple procyanidin oligomers (monomer through pentamer) was achieved by isocratic elution of hexane–methanol–ethyl acetate over a silica based columns (Yanagida et al. 2000). Modifications of Rigaud's method (Rigaud et al. 1993) by Hammerstone et al. (1999) resulted in improved separation of cocao procyanidin oligomers up to decamer. In another study, Adamson et al. (1999) purified cocoa procyanidin oligomers for quantification of procyanidins in various chocolate and chocolate liquors. Alternatively, Waterhouse et al. (2000) used a formula based on cocoa procyanidin monomer, oligomers and polymer retention times and number of subunits to describe amounts of monomer, oligomers and polymer in the more complicated grape seed extract. More recently, procyanidin monomer through decamer and polymer were quantified in cocoa, brown sorghum bran, cranberry, and blueberry using purified cocoa oligomers and polymers (Gu et al. 2002). Hammerstone et al. (1999) coupled his HPLC method with API–MS to provide additional qualitative information regarding molecular characteristics of higher oligomeric procyanidins (Hammerstone et al. 1999). The HPLC–API–MS method has been applied qualitatively to a variety of other foods and beverages including peanut, tea, almonds, wine, grape juice, apple, cinnamon (Lazarus et al. 1999), blueberry and cranberry (Prior et al. 2001). Matrix assisted laser desorption ionization–time of flight–mass spectrometry (MALDI–TOF–MS) has been used in the identification of catechin oligomers up to pentadecamer in apple (Guyot et al. 1997) and up to nonamer in grape seeds (Yang and Chien 2000) however, these methods were not quantitative.

Utilization of mass spectrometers for the unequivocal quantification of higher oligomers necessitates the use of pure standards. Unfortunately, oligomers beyond trimer are commercially unavailable. Isolation or synthesis of standards are options however with the exception of some laboratories, are not feasible due to lack of time and expertise. Typically, the researcher would have to make the assumptions that molar ionization efficiencies for all oligomers are identical. Quantification would then be based on catechin equivalent units much in the same way colorimetric or antioxidant assays base their results.

### *Electrochemical detection methods*

HPLC–electrochemical detection (ED) of proanthocyanidins (Chiavari et al. 1987, McMurrough and Madigan 1996; Whittle et al. 1999) and flavonoids

(Jungbluth and Ternes 2000) has become more prevalent since these compounds are quite electroactive thus offering a degree of selectivity and sensitivity not obtainable with UV or FLD. Coulometric electrochemical detectors, composed of a series of electrodes set at different potentials can provide additional qualitative information with regards to different hydroxyl substitution patterns on proanthocyanidins (ESA—Detection of Free Proanthocyanidins).

### Antioxidant methods of analysis

### *Antioxidant activity*

Proanthocyanidins, as well as other phenolics are potent biological antioxidants because of their favorable redox-potentials, free radical scavenging activity and thus their ability to form less reactive phenoxyl radicals. Numerous assays exist for the measurement of antioxidant activity in plasma and in various foods and beverages. Several assays routinely conducted include; oxygen radical absorbant capacity (ORAC), trolox equivalent antioxidant capacity (TEAC), ferric reducing/antioxidant power (FRAP), and total radical-trapping antioxidant parameter (TRAP) assays. Discussions regarding assays with biological endpoints such as oxidative DNA damage, LDL damage, and reactive nitrogen species is presented elsewhere (Shi et al. 2001). Of these assays, the ORAC assay tends to be the preferred method to determine antioxidant capacity in foods and as an indicator for potential biological activity.

The ORAC assay has been widely applied to measure *in vitro* and *in vivo* antioxidant activity. The ORAC assay provides a means to measure antioxidant activity of various plant extracts, pure compounds and plasma. Complete antioxidant protection level in plasma and potential risk of developing age related degenerative diseases have been determined by ORAC. Unlike, TEAC, FRAP and TRAP assays, ORAC uniquely measures both inhibition time and inhibition degree—which better represents overall antioxidant activity since reaction is driven to completion. With ORAC, 2, 2′-azobis(2- amidino-propane) (AAPA) is used to generate free radicals, then free radical damage is assessed with a fluorescent probe (i.e. β-phycoerythrin) and represented by a decrease in fluorescence intensity. When a phenolic antioxidant is present, the fluorescent probe is preserved and this is indicative of the phenolic antioxidant capacity or ability to protect the probe from free radical damage. Ou et al. (2001, 2002) reported an improved ORAC assay that replaces β-phycoerythrin with fluorescein as a new fluorescent probe. Problems with original method (Cao et al. 1993) included photo bleaching of β-phycoerythrin and complexation between β-phycoerythrin and proanthocyanidins were overcome with this advancement. Authors of the improved method also provide detail on merits of this assay over other commonly used assays (TEAC, FRAP, and TRAP). More recently, a high throughput fully automated ORAC assay was developed that utilizes 96-well microplates (Huang et al. 2002). Prior to this development, the ORAC assay was limited to laboratories possessing the discontinued COBAS FAR II instrument.

The ORAC assay described above is useful in determination of antioxidant activity for many tested compounds. However, is this data biologically relevant? Biological relevance of *in vitro* data comes into question since only intact compounds have been studied. The metabolic fate of many flavonoids can involve deglycosylation, methylation, sulphation, glucuronidation, and degradation into simpler phenolics (Rice-Evans et al. 2001). These modifications in structure will dictate absorbtion and where they function *in vivo*. The lack of analyses of modified flavonoids is in part due to an incomplete understanding of the metabolic fate for a given compound. However, for the known flavonoid metabolites, it is a lack of commercial standards that impedes research in this area. Consequently, some laboratories have synthesized the 3′ and 4′ methyl ethers of (+) catechin and (−) epicatechin for use in HPLC method development (Donovan et al. 1999).

Ascorbate and vitamin E (typically, TROLOX a water-soluble form of Vitamin E) are often selected because they are considered the gold standard for antioxidant assays to which other compounds are compared. Speculation based on *in vitro* data where TROLOX is a standard for *in vivo* importance of a given flavonoid is problematic due to the fact that vitamin E is lipophilic whereas flavonoids are not—the *in vivo* sites of possible function would be different.

### Analytical standards

Availability of more standards to analytical chemists would lead to better analytical methods critical to scientists from a wide variety of disciplines. Preparation of analytical standards is expensive and laborious. The most common method of standard preparation involves isolation from crude plant extracts. Most commercially available standards are obtained in just such a manner. Standards could be prepared via alternative methods such as plant tissue culture or biotransformation. However, these methods are limited in their application. Complete organic synthesis is another possibility, however synthesis can be quite challenging especially in the synthesis of flavonoids and proanthocyanidins oligomers. The production of $^{2}H$- and $^{13}C$-labeled phenolics would provide the analytical chemist with ideal standards that would behave nearly identical to natural forms in terms of MS ionization efficiencies. Despite synthetic challenges, several groups (Arnaudinaud et al. 1998, Caldwell et al. 2000, Kozikowski et al. 2000) have demonstrated the successful synthesis of flavonoids and procyanidins. Kozikowski et al. (2000) described the synthesis of procyanidin B2 dimer, however rather low yields were obtained. The synthesis of $^{13}$C-labeled quercetin 4′-*O*-β-D-glucoside was accomplished for future studies examining the biological fate of this flavonol in rats (Caldwell et al. 2000). The synthesis of a $^{13}$C-labeled chalcone, a key intermediate in the synthesis of procyanidins has been demonstrated (Arnaudinaud et al. 1998). Research previously conducted using several analytical methods was used to study the metabolism of $^{2}$H- and $^{13}$C-labeled carotenoids (Pawlosky et al. 2000, Wang et al. 2000, Yao et al. 2000). Analogous projects with isotopically labeled flavonoids and proanthocyandins would be of great value to human nutrition investigations and results from such research would provide better insight on the absorption, bioavailability, and metabolism of flavonoids.

### Conclusions

Flavanols and proanthocyanidins are attracting tremendous interest in field of health and nutrition, especially with regard to their potential cardiovascular health benefits. Given their ubiquitous occurrence in plant-based foods, it is critical that appropriate analytical methodologies be developed and used to characterize and quantify at least the major flavanols and proanthocyanidins present in commonly consumed foods and beverages.

At present, a variety of analytical assays and methods are used to assess the levels of flavanols and proanthocyanidins in foods and beverages, with many of these being wholly inadequate. In particular, non-specific assays can completely misrepresent the true flavanol and proanthocyanidin content of many foods and beverages, and therefore results from studies utilizing these assays should be interpreted with great caution. The development of increasingly robust and sensitive HPLC methodologies has made possible the construction of useful databases for many classes of phytochemicals, and just such a database is under development at present. However, the most significant limitation to constructing more comprehensive phytochemical food and beverage composition databases is the general lack of appropriate standards. Indeed, the lack of all but a limited number of flavanol and proanthocyanidin standards will limit the number of compounds that can be reliably included in this initial database. Nevertheless, it is possible to generate reliable food and beverage composition data for a limited number of individual flavanols and proanthocyanidins, including many of those specifically associated with potential cardiovascular health benefits. Going forward, it will be essential for investigators to use appropriate analytical methodologies to properly characterize the contribution of dietary flavanols and proanthocyanidins to human health. In addition, it will be important for the analytical and nutrition research communities to collaborate in a multi-disciplinary manner to facilitate the development of increasing number of reliable analytical standards so that the full extent of the health effects of these fascinating phytochemicals can be fully understood.

### References

Adamson GE, Lazarus SA, Mitchell AE, et al. 1999. HPLC method for the quantification of procyanidins in cocoa and chocolate samples and correlation to total antioxidant activity. J Agric Food Chem 47:4184–4188.

Arnaudinaud V, Nguyen L, Nay B, Peyrat J.F, Cheze C, Vercauteren J. 1998. Citrus flavonoids as starting material for total synthesis of condensed tannins. *2nd International Electronic Conference on Synthetic Organic Chemistry (ECSOC-2)*. http://www.mdpi.org/ecsoc/, September 1–30.

Bramley PM. 2002. Is lycopene beneficial to human health? Phytochemistry 54:233–236.

Caldwell ST, Crozier A, Hartley RC. 2000. Isotopic labeling of quercetin 4′-*O*-β-D-glucoside. Tetrahedron 56:4101–4106.

Cao G, Alessio HM, Cutler R. 1993. Oxygen-radical absorbance capacity assay for anti-oxidants. Free Radic Biol Med 14:303–311.

Carando S, Teissedre PL, Rascual-Martinez L, Cabanis JC. 1999. Levels of flavan-3-ols in French wine. J Agric Food Chem 47:4161–4166.

Chandra A, Rana J, Li Y. 2001. Separation, identification, quantification, and method validation of anthocyanins in botanical supplement raw material by HPLC and HPLC–MS. J Agric Food Chem 49:3515–3521.

Chiavari G, Vitali P, Galletti GC. 1987. Electrochemical detection in the high-performance liquid chromatography of polyphenols (vegetable tannins). J Chromatogr 392:426–434.

Dawson MI. 2000. The importance of vitamin A in nutrition. Curr Pharm Des 6:311–325.

Donovan JL, Luthria DL, Stremple P, Waterhouse AL. 1999. Analysis of (+)-catechin, (−)-epicatechin and their 3′- and 4′-*O*-methylated analogs. A comparison of methods. J Chromatogr B 726:277–283.

Drewnowski A, Gomez-Carneros C. 2000. Bitter taste, phytonutrients, and the consumer: A review. Am J Clin Nutr 72:1424–1435.

ESA—Detection of Free Proanthocyanidins, Application Note 5600, ESA, Inc. 22 Alpha Road, Chelmsford, MA 01824-4171 USA.

Fuleki T, Ricardo da Silva JM. 1997. Catechin and procyanidin composition of seeds from grape cultivars grown in Ontario. J Agric Food Chem 45:1156–1160.

Graham HD. 1992. Stabilization of the Prussian blue color in the determination of polyphenols. J Agric Food Chem 40:801–805.

Grayer RJ, Kite GC, Abuo-Zaid M, Archer LJ. 2000. The application of atmospheric pressure chemical ionization liquid chromatography-mass spectrometry in chemotaxinomic study of flavonoids: characterization of flavonoids from *Ocimum gratissimum* var. *gratissimum*. Phytochem Anal 11:257–267.

Gu L, Kelm M, Hammerstone JF, et al. 2002. Fractionation of polymeric procyanidins from lowbush blueberry and quantification of procyanidins in selected foods with an optimized normal-phase HPLC–MS fluorescent detection method. J Agric Food Chem 50:4852–4860.

Guyot ST, Doco T, Souquet JM, Moutounet M, Drilleau JF. 1997. Characterization of highly polymerized procyanidins in apple cider (*Malus sylvestris* var. *kermerrien*) skin and pulp. Phytochemistry 44:351–357.

Hagerman, A. (2002) Tannin Chemistry. http://www.users.muohio.edu/hagermae/tannin.pdf (Accessed November 2002).

Hammerstone JF, Lazarus SA, Mitchell AE, Rucker R, Schmitz HH. 1999. Identification of procyanidins in cocoa and chocolate by high performance liquid chromatography/mass spectrometry. J Agric Food Chem 47:490–496.

Hertog MG, Kromhout D, Aravanis C, et al. 1995. Flavonoid intake and long-term risk of coronary heart disease and cancer in seven countries study. Arch Intern Med 155:381–386.

Huang D, Ou B, Hampsch-Woodill M, Flanagan JA, Prior RL. 2002. High-throughput assay of oxygen radical absorbance capacity (ORAC) using a multichannel liquid handling system coupled with a microplate fluorescence reader in 96-well format. J Agric Food Chem 50:4437–4444.

Jungbluth G, Ternes W. 2000. HPLC separation of flavonols, flavones and oxidized flavonols with UV-, DAD-, electrochemical and ESI-ion trap MS detection. Presenius J Anal Chem 367:661–666.

Kerchesy JJ, Hemingway RW. 1986. Condensed tannins: (4 beta to 8;2 β to *O* to 7)-linked procyanidins in *Arachis hypogea* L. J Agric Food Chem 34:966–970.

Knekt P, Kumpulainen J, Jarvinen R, et al. 2002. Flavonoid intake and risk of chronic disease. Am J Clin Nutr 76:560–568.

Kozikowski AP, Tuckmantel W, George C. 2000. Studies in polyphenol chemistry and bioactivity. 2. Establishment of interflavan linkage regio- and stereochemistry by oxidative degradation of an *O*-alkylation derivative of procyanidin B2 to (R)-(−)-2,4-diphenylbutyric acid. J Org Chem 65:5371–5381.

Lazarus SA, Adamson GE, Hammerstone JF, Schmitz HH. 1999. High-performance liquid chromatography/mass spectrometry analysis of proanthocyanidins in foods and beverages. J Agric Food Chem 47:3693–3701.

Lou H, Yamazaki Y, Sasaki T, Uchida M, Tanaka H, Oka S. 1999. A-t proanthocyanidins from peanut skins. Phytochemistry 51:297–308.

Matthews S, Mila I, Scalbert A, et al. 1997. Extractable and non-extractable proanthocyanidins in barks. J Agric Food Chem 45:1195–1201.

McMurrough I, Madigan D. 1996. Semipreparative chromatographic procedure for the isolation of dimeric and trimeric proanthocyanidins from barley. J Agric Food Chem 44:1731–1735.

Merken HM, Beecher GR. 2000. Measurement of food flavonoids by high-performance liquid chromatography: A review. J Agric Food Chem 48:577–599.

Merken HM, Merken CD, Beecher GR. 2001. Kinetics method for the quantitation of anthocyanidins, flavonols, and flavones in foods. J Agric Food Chem 49:2727–2732.

Ou B, Hampsch-Woodill M, Prior RL. 2001. Development and validation of an improved oxygen radical absorbance capacity assay using fluorescein as the fluorescent probe. J Agric Food Chem 49:4619–4626.

Ou B, Hampsch-Woodill M, Flanagan J, Deemer EK, Prior RL, Huang D. 2002. Novel fluorometric assay for hydroxyl radical prevention capacity using fluorescein as the probe. J Agric Food Chem 50:2772–2777.

de Pascual-Teresa, S., Medez-Arroyo, A., Santos -Buelga, C., Rivas-Gonzalo, J.C. (1998) "Determination of flavan-3-ols in some Spanish wines". In: *Polyphenol Communications 98*, XIXth International Conference on Polyphenols Lille (France), 333–334.

de Pascual-Teresa S, Santos-Buelga C, Rivas-Gonzalo JC. 2000. Quantitative analysis of flavan-3-ols in Spanish foodstuffs and beverages. J Agric Food Chem 48:5331–5337.

Pawlosky RJ, Flanagan VP, Novotny JA. 2000. A sensitive procedure for the study of β-carotene-$d^8$ metabolism in humans using high performance liquid chromatography–mass spectrometry. J Lipid Res 41:1027–1031.

Peng Z, Hayasaka Y, Iland PG, Sefton M, Hoj P, Waters EJ. 2001. Quantitative analysis of polymeric procyanidins (tannins) from grape (*Vitis vinifera*) seeds by reverse phase high-performance liquid chromatography. J Agric Food Chem 49:26–31.

Porter LJ. 1994. The flavonoids: advances in research since 1980. In: Harbourne JB, editor. Flavans and proanthocyanidins. London, UK: Chapman and Hall. p 23–53.

Price ML, Butler LG. 1977. Rapid visual estimation and spectrophotometric determination of tannin content of sorghum grain. J Agric Food Chem 25:1268–1273.

Prior RL, Lazarus SA, Cao G, Muccititelli H, Hammerstone JF. 2001. Identification of procyanidins and anthocyanidins in blueberries and cranberries (*Vaccinium* spp.) using high-performance liquid chromatography/mass spectrometry. J Agric Food Chem 49:1270–1276.

Rice-Evans C. 2001. Flavonoid antioxidants. Curr Med Chem 8:797–807.

Rigaud J, Escribano-Bailon MT, Prieur C, Souquet JM, Cheynier V. 1993. Normal-phase high-performance liquid chromatographic separation of procyanidin from cacao beans and grape seeds. J Chromatogr A 654:255–260.

Santo-Buelga C, Scalbert A. 2001. Proanthocyandins and tannin-like compounds—nature, occurrence, dietary intake and effects on nutrition and health. J Sci Food Agric 80:1094–1117.

Shahidi F, Naczk M. 1995. Methods of analysis and quantification of phenolic numbers. In: Food phenolics, sources, chemistry, effects, applications. Technomic Publishing Company, Inc. p 295.

Shi H, Noguchi N, Niki E. 2001. Galvinoxyl method for standardizing electron and proton donation activity. In: Packer L, editor. Methods in Enzymology. 335. Academic Press. p 157–308.

Swain T, Hillis WEJ. 1959. Phenolic constituents of Prunus domestica I. Quantitative analysis of phenolic constituents. J Sci Food Agric 10:63.

Treutter D. 1990. A new HPLC technique with a selective chemical reaction detection of catechins and condensed tannins. Planta Med 56:578.

Wang Y, Xu X, van Lieshout M, et al. 2000. A liquid chromatography–mass spectrometry method for the quantification of bioavailability and bioconversion of β-carotene to retinol in humans. Anal Chem 72:4999–5003.

Waterhouse AL, Ignelzi S, Shirley JR. 2000. A comparison of methods for quantifying oligomeric proanthocyanidins from grape seed extracts. Am J Enol Vitic 51:383–389.

Whittle N, Eldridge H, Bartley J. 1999. Identification of the polyphenols in barley and beer by HPLC/MS and HPLC/electrochemical detector. J Inst Brew 105:89–99.

Wilson EL. 1979. High-pressure liquid chromatography of apple juice phenolic compounds. J Sci Food Agric 30:255–260.

Wollgast J, Pallaroni L, Agazzi M, Anklam E. 2001. Analysis of proanthocyanidins in chocolate by reverse-phase high-performance chromatography with electrospray ionisation mass spectrometric and tandem mass spectrometric detection. J Chromatogr A 926:211–220.

Yanagida A, Kanda T, Takashida T, Kamimura A, Hamazono T, Honda S. 2000. Fractionation of apple procyanidins according to their degree of polymerization by normal-phase high-performance liquid chromatography. J Chromatogr A 890:251–259.

Yang Y, Chien M. 2000. Characterization of grape procyanidins using high-performance liquid chromatography/mass spectrometry and matrix-assisted laser desorption/ionization time-of-flight mass spectrometry. J Agric Food Chem 48: 3990–3996.

Yao L, Liang Y, Trahanovsky WS, Serfass RE, White WS. 2000. Use of a $^{13}C$ tracer to quantify the plasma appearance of a physiological dose of lutein in humans. Lipids 35:339–348.

Zeeb DJ, Nelson BC, Albert K, Dalluge JJ. 2000. Separation and identification of twelve catechins in tea using liquid chromatography/atmospheric pressure chemical ionization-mass spectrometry. Anal Chem 72:5020–5026.

